# Flowers and Leaves Extracts of *Stachys palustris* L. Exhibit Stronger Anti-Proliferative, Antioxidant, Anti-Diabetic, and Anti-Obesity Potencies than Stems and Roots Due to More Phenolic Compounds as Revealed by UPLC-PDA-ESI-TQD-MS/MS

**DOI:** 10.3390/ph15070785

**Published:** 2022-06-23

**Authors:** Sabina Lachowicz-Wiśniewska, Anubhav Pratap-Singh, Ireneusz Kapusta, Angelika Kruszyńska, Andrzej Rapak, Ireneusz Ochmian, Tomasz Cebulak, Wioletta Żukiewicz-Sobczak, Paweł Rubiński

**Affiliations:** 1 Department of Food and Nutrition, Calisia University, 4 Nowy Świat Street, 62-800 Kalisz, Poland; wiola.zukiewiczsobczak@gmail.com (W.Ż.-S.); p.rubinski@akademiakaliska.edu.pl (P.R.); 2 Department of Horticulture, West Pomeranian University of Technology in Szczecin, 71-434 Szczecin, Poland; ireneusz.ochmian@zut.edu.pl; 3 Faculty of Land and Food Systems (LFS), The University of British Columbia, Vancouver Campus 213-2205 East Mall, Vancouver, BC V6T 1Z4, Canada; anubhav.singh@ubc.ca; 4 Department of Food Technology and Human Nutrition, College of Natural Science, Rzeszow University, 4 Zelwerowicza Street, 35-601 Rzeszow, Poland; ikapusta@ur.edu.pl (I.K.); tomcebulak@gmail.com (T.C.); 5 Laboratory of Tumor Molecular Immunobiology, Ludwik Hirszfeld Institute of Immunology and Experimental Therapy, Polish Academy of Sciences, 53-114 Wroclaw, Poland; angelika.kruszynska@hirszfeld.pl (A.K.); andrzej.rapak@hirszfeld.pl (A.R.)

**Keywords:** bioactive compounds, in vitro biological potency, medical plant, marsh woundwort

## Abstract

The present work aims to assess the biological potential of polyphenolic compounds in different parts (flowers, leaves, stems, and roots) of *Stachys palustris* L. Towards secondary metabolites profile, 89 polyphenolic compounds (PCs) were identified by UPLC-PDA-ESI-TQD-MS/MS, with a total average content of 6089 mg/100 g of dry matter (d.m.). In terms of biological activity, antioxidant activity (radical activity, reducing power), digestive enzyme inhibitory (α-glucosidase, α-amylase, pancreatic lipase) effect, and antiproliferative activity (inhibition of cell viability and induction of apoptosis in different human cancer cell lines) were explored. Leaves, flowers, stems, and roots of *S. palustris* L. have not been studied in this regard until now. Vescalagin and cocciferin d2, isoverbascoside (verbascoside), luteolin 6-*C*-glucoside, luteolin 6-*C*-galactoside, apigenin 6-*C*-glucoside, (−)-epicatechin, ellagic acid, and malvidin 3-*O*-diglucoside were detected as main ingredients in the studied parts. Methanolic extract of *S. palustris* L. leaves and flowers revealed the highest amount of PCs with the strongest antiradical (18.5 and 15.6 mmol Trolox equivalent (TE)/g d.m., respectively) and reducing power effects (7.3 and 5.6 mmol TE/g d.m.). Leaf extracts exhibited better α-amylase and pancreatic lipase inhibition effects, while flower extracts exhibited better α-glucosidase inhibition effect. Regarding antiproliferative activity, extracts of the leaves and flowers significantly reduced cell viability and induced a high level of apoptosis in human lung, pancreatic, bladder, and colon cancer cell lines, as well as in human acute myeloid leukemia; whereas the extracts from stems and roots revealed the weaker effects. The results of this work showed anti-proliferative and potentially anti-diabetic, anti-obesity properties of *S. palustris* L., especially for flowers and leaves, which may have wide potential applications in the functional food, special food, pharmaceutical, cosmetics industries, and/or in medicine.

## 1. Introduction

In recent years, edible plants, forgotten plants, wild plants, as well as medical plants have emerged as the potential sources of secondary metabolites for therapeutic interventions [1], which has opened doors for their use as active ingredients in food, pharmaceutical, cosmetics, and/or medical products.

*Stachys palustris* L. (sect. Stachys; family Lamiaceae, subfamily Lamioideae), otherwise known as marsh woundwort, is one such edible plant found in both lowlands and mountains, which has been used in traditional medicines, such as emetic, antiseptic, nervine, sedative, antispasmodic, emmenagogue, hemostatic, vulnerable, expectorant, and tonic agent [2,3,4,5]. However, it is most effective in treating internal and external hemorrhages, cramps, and joint pains, as well as the treatment of gout [2]. This plant can grow up to 1 m tall, has square stems, purplish-red flowers arranged in whorls, opposite leaves, and dry four-chambered schizocarp fruit [2].

The biosynthesis of polyphenolic compounds is an intensive process, largely conditional on abundant factors linked with the plant and their environment. The distribution and concentration of bioactive compounds in different underground and above-ground parts of the plant can be strongly varied. This is mainly tied to the role of polyphenols in the growth phase and in plants’ life cycle [6]. Previous research has shown that *S. palustris* L. plants exhibit a high total polyphenol content of about 213 mg gallic acid equivalent (GAE)/g dry matter (d.m.), high antiradical activity against DPPH radical, and promising anti-proliferative properties against cervix adenocarcinoma cells (HeLa) [2,7]. On the other hand, the chromatographic analysis showed the presence of only eight compounds belonging to the group of phenylethanoid glycosides, isoscutellarein derivatives, phenolic acid, and iridoids [2].

Despite the above data and traditional knowledge about *S. palustris* L. being a high nutraceutical potential, little is known about the secondary metabolites profile and biological activity of edible leaves, flowers, stems, and roots of *S. palustris* L. [6,7]. Therefore, this study aimed to assess the profile and amount of polyphenolic compounds by UPLC-PDA-ESI-TQD-MS/MS and their in vitro biological activity (antioxidant and anti-proliferative activity). Results, on the polyphenol profile and anti-proliferative activity, as well as inhibitory activity against digestive enzymes, could be interesting for potential applications in functional foods, natural health products, pharmaceuticals, cosmetics industries, and/or medicine.

## 2. Results and Discussion

### 2.1. Profile of Polyphenolic Compounds

The UPLC–PDA-ESI–TQD–MS/MS fingerprint of *Stachys palustris* L. flowers, leaves, stems and roots (Table 1, and Figure S1) discovered the presence of 89 polyphenolic ingredients based on their UV maxima, *m*/*z*, [M-H]^−^ and [M-H]^+^, retention times, peak areas, available data, and standards. Amongst identified compounds, 40 hydrolysable tannins, 16 phenylethanoid glycosides (PhGs), 4 anthocyanins, 1 flavan-3-ol, 6 phenolic acids, 1 flavonols, and 21 flavones were found. However, the qualitative composition of the tested bioactive ingredients was strongly dependent on the extracted part of the plant; with 56, 55, 50, and 49 compounds identified, respectively, in flowers, roots, leaves, and stems of *S. palustris* L (Table 1 and Table 2). It is worth emphasizing that thus far only eight compounds had been identified in *S. palustris* L. belonging to secondary metabolites, such as verbascoside, echinacoside, two isoscutellarein derivatives, monomelittoside, chlorogenic acid, harpagide, and its derivative 8-*O*-acetyl-harpagide [2]. However, in our study, compounds, such as monomelittoside, chlorogenic acid, harpagide, and its derivative 8-*O*-acetyl-harpagide [2], were not observed. Thus, apart from the compounds confirmed by Venditti et al. [2], the remaining compounds were detected in the flowers, leaves, stems, and roots extracts of *S. palustris* L. for the first time.

#### 2.1.1. Hydrolysable Tannins

The main class of polyphenolic compounds identified in the flowers, leaves, stems, and roots of *S. palustris* L. was hydrolysable tannins. In this study, *S. palustris* L. was found to contain 40 compounds of this class, including 36 hydrolysable tannins in the flowers, 31 in the leaves, 32 in the stems, and 22 in the roots. This group contains the derivatives of gallic acid, ellagitannins, and gallotannins [8]. Gallic acid is known to dimerize into ellagic acid formed by intramolecular dilactonization of HHDP acid (hexahydroxydiphenoyl acid). Ellagitanins, constituting the most abundant fraction of hydrolysable tannins, are formed by the oxidative coupling of adjacent galloyl fractions from gallotannins [8,10]. The group of HHDP is further divided into two groups: a NHTP (nonahydroxytriphenoyl) group by coupling to the next galloyl group, or a Chebuloyl group by double oxidation [8,10]. The structure of thirty-four compounds were confirmed, based on identification of their fragmentation pattern on the basis of fragmentation earlier described in the Chestnut of *Castanea sativa* by Miller [8], the different plant parts of *Terminalia arjuna* [9], the fraction of *Sanguisorba officinalis* L. [11], the different parts of myrtle berry from Italy [12], *Fragaria vesca* L. berries [13], *Terminalia chebula* fruits [14], plant medical [15,16], Myrtaceae family [17], *Genista tinctoria* L. [18], and the leaves of *Phyllagathis rotundifolia* [19]. These compounds were identified as grandinin, two grandinin derivatives, roburin E (*m*/*z* 1065), fourteen castalagin/vescalagin isomers (*m*/*z* 933), four pedunculagin isomer [diHHDP-glucose] (*m*/*z* 783), two casuarinin [diHHDP-galloyl-glucose] (*m*/*z* 935), casuarictin [galloyl-DiHHDP-glucose] (*m*/*z* 935), two chebulanin (*m*/*z* 651), geraniin isomer (*m*/*z* 951), pentagalloyl-glucose (*m*/*z* 939), two tellimagrandin I [digalloyl-HHDP-glucose] (*m*/*z* 785), and four trigalloyl-HHDP-glucose (*m*/*z* 937).

The fragmentation of the above hydrolysable tannins resulted in losses of typical residues such as gallic acid ([M-H-170]^−^), galloyl ([M-H-152]^−^), HHDP ([M-H-302]^−^), HDDP glucose ([M-H-482]^−^), galloyl-HDDP-glucose ([M-H-634]^−^), or galloyl-glucose ([M-H-332]^−^) residues [8,10,11]. According to Esposito et al. [8], the compound of chebulagic acid (galloylo-chebuloyl-HHDP-glucose) contains chebuloyl group generated by the oxidation of HHDP residues and further supported by the loss of carboxylic and chebuloyl groups. In addition, the compound cocciferin d2 isomer (HHDP-NHTP-glucose-galloyl-diHHDP-glucose), which is a dimer ellagitannin, was noted as [M-2H]^2−^ (double charged pseudomolecular ion). Sanguiin H-10 isomers [M-H]^−^ at *m*/*z* 1567 were identified on the basis of data published by Kool et al. [20] in Boysenberry *(Rubus loganbaccus × baileyanus* Britt.), and by Mullen et al. [21] during the fragmentation MS/MS loss of two HHDP (302 mass unit (m.u.), glucosyl (162 m.u.), and galloyl (170 m.u.) moieties.

#### 2.1.2. Flavanones

The compounds belonging the group of flavones usually subsist as glycosides and seldom as free aglycones [37]. In the full-scan LC-MS/MS, the deprotonated pseudomolecular ions [M-H]^−^ of luteolin, apigenin, isoscutellarein, and chrysoeriol (*m*/*z* 285.0, *m*/*z* 269.0, *m*/*z* 285.0, and *m*/*z* 299, respectively) were observed [33]. A total of 21 compounds are reported in different parts of *S. plasturis* L., including 7 flavones in the flowers, 9 in the leaves, 8 in the stems where apigenins and luteolins dominated, and 15 in the roots where isoscutellarein and chrysoeriol dominated. The pathway for flavones *C*-glycosides showed the decomposition of glycan with a loss of neutral residues with the decomposition of a flavonoid moiety associated to the residual part of glycan [38]. These compounds for the genus *Stachys* are considered important chemosystematics markers [25].

The first class of flavones was luteolin ([M-H]^−^ at *m*/*z* 447) determined based on characteristic UV absorption maxima at 269 and 349 nm. According to Lin et al. [37], these compounds are characterized by two UV spectral maximums: 250–300 nm (band I), and 342–350 nm (band II). Based on the fragmentation of the peak that indicated the presence of a *C*-glycosylated derivative, these compounds were confirmed as luteolin 6-*O*-galactoside and luteolin 6-*O*-glucoside (previously identified in the *Elaeis guineensis* Jacq.) [33,34].

Apart from luteolins, four apigenin compounds (*m*/*z* 431, 577, and 635) were also identified based on the UV spectrum at 260 and 336 nm. Two compounds with molecular ion at *m*/*z* 431 and the fragmentation of MS/MS indicative of a *C*-glycosylated derivative and loss of glucosyl (162 m.u.) moiety were identified as apigenin 6-*C*-galactoside and apigenin 6-*C*-glucoside, previously noted in *Cyclanthera pedata* leaves, fruits, and dietary supplements [35]. The compound with pseudomolecular ion at *m*/*z* 635 was tentatively identified on the basis of fragmentation earlier described in *Stachys parviflora* L. [26] as apigenin acetylallosyl-glucoside. Mass spectra of apigenin with *p*-coumaric acid derivative showed molecular ion at *m*/*z* 577 and was confirmed based on previous results for *Stachys byzantina* [36] and *Stachys bombycina* [31] as apigenin 7-*O*-*β-D-*(6-*p*-coumaroyl)-glucopyranoside.

Two compounds belonging to chrysoeriol derivatives with pseudomolecular ion at *m*/*z* 667 and *m*/*z* 677 were confirmed based on the fragmentation pathway described in the *Stachys subgenus* [32] and *Stachys bombycina* [31] and were identified as chrysoeriol 7-*O*-acetylallosylglucoside and chrysoeriol-acetyl-allopyranosyl-glucopyranoside.

The last class of flavones were twelve isoscutellarein derivatives with the pseudomolecular ion [M-H]^−^ *m*/*z* 667, *m*/*z* 707, *m*/*z* 665, and *m*/*z* 651. The presence of Aglycon [A-H]^−^ fragment and *D*-allose, which is characteristic of a large group of *Stachys* plants, was noted [25]. As constituents acetylated on the internal or external allose unit showing the intermediate ions [M-180]^−^ or [M-180-OAc]^−^ [25], these compounds were identified as isoscutellarein-acetylallosyl-(glucopyranoside)apiose isomers (nine compounds), 4′-*O*-methylisoscutellarein-acetyl-allosyl-glucopyranoside, and their isomers similar to *Stachys recta* L. [25] and *Stachys parviflora* L. [26].

#### 2.1.3. Phenylethanoid Glycosides (PhGs)

Phenylethanoid glycosides (PhGs) are appreciated for a strong biological potency [39,40]. The typical spectra exhibited UV-Vis maxima between 320–340 nm [39]. 16 PhG compounds, including 2 in the flowers, 3 in the leaves, 2 in the stems, and 13 in the roots, were detected in *S. palustris* L. The detected PhGs showed deprotonated molecular ions [M-H]^−^ at *m*/*z* 785, *m*/*z* 755, *m*/*z* 623, *m*/*z* 799, *m*/*z* 769, *m*/*z* 651, *m*/*z* 755, and *m*/*z* 669. During the fragmentation of the above, PhGs showed losses of typical residues, such as the neutral loss of the caffeoyl residue (162 m.u.), *p*-coumaric moiety (176 m.u.), the rhamnose residue (146 m.u.), the glucose residue (162 m.u.), the COCH_2_ group (42 m.u.), a CH_2_ radical (14 m.u.), and the methoxy group (30 m.u.) [25,26,39,40].

Compounds with pseudomolecular ions at *m*/*z* 785, *m*/*z* 623, *m*/*z* 755, and *m*/*z* 669 were confirmed as betonyoside E, two B-OH-Forsythoside B methylether isomers, isoacteoside, two forsythoside B, and their isomer based on stachysoside E, previously isolated from *Stachys recta* L. [25], *Stachys parviflora* L. [26], *Stachys officinalis* L. [27], and *Stachys alopecuros* L. [2].

Mass spectra for two compounds were tentatively assigned as echinacoside (*m*/*z* 799) and cistanoside A (*m*/*z* 785), previously described in *Cistanche deserticola* [22] and *Cistanche armena* [23].

Three compounds with pseudomolecular ions at *m*/*z* 756 and 637 was tentatively detected as two stachysoside A and leucoseptoside A, with a characteristic fragmentation pathway presented in *Lagopsis supina* [28].

The last three compounds belonging to PhGs were tentatively specified on the basis [24] noted in *Phlomis* species. Two compounds having pseudomolecular ions at *m*/*z* 769, and on the basis of fragmentation MS/MS the losses of *p*-coumaric residue, pentose, and glucose moiety, were tentatively assigned as alyssonoside and alyssonoside isomer [24]. Compounds, such as samioside (*m*/*z* 755) and martynoside (*m*/*z* 651), were assigned on the losses of glucose, pentose, rhamnose moiety, and *p*-coumaric and rhamnose moiety, according to the fragmentation pattern determined by Kirmizibekmes et al. [24].

#### 2.1.4. Other Phenolic Acids, Anthocyanins, Flavonol, and Flavan-3-ol

The last groups of polyphenolic compounds identified in the different parts of *S. palustris* L. were phenolic acids, anthocyanins, flavonol, and flavan-3-ol. Among detected phenolic acids, three compounds presented similar maximum absorbance at 325 nm, which is typical for derivatives of dicaffeoylquinic acid [25]. All of them had common pseudomolecular ions at *m*/*z* 515. The first compound was detected as 3,5-di-affeoylquinic acid compared with the standard. The next two compounds noted the characteristic ions at *m*/*z* 173, which indicate the presence of quinic acid in position four. Thus, these compounds were determined as 3,4-di-caffeoylquinic and 4,5-di-caffeoylquinic acids, respectively, on the basis of the order of elution [25]. The next three phenolic acid tentatively assigned as ellagic acid (*m*/*z* 301), ellagic acid glucoside ([M-H-463]^−^), and ellagic acid pentoside ([M-H-461]^−^) were previously determined in *Stachys officinalis* [33].

All anthocyanins and flavonol assigned in *S. palustris* L. was noted in just flowers for the first time. In the full-scan LC-MS/MS, the deprotonated molecular ions [M-H]^−^ of delphinidin, malvidin, and cyanidin (*m*/*z* 303, *m*/*z* 331, and *m*/*z* 287, respectively) [29] were detected. These compounds were detected as delphinidin 3-*O*-glucoside, malvidin 3-*O*-diglucoside, cyanidin 3-*O*-glucoside, and malvidin 3-*O*-acetylglucoside on the basis of their fragmentation pathways [29]. One flavonol (deprotoned molecular ion at *m*/*z* 285) was specified as kaempferol hexose glucuronide from the losses of hexose moiety (162 m.u.) and glucuronide moiety (176 m.u.) [30]. The existence of (−)-epicatechin was confirmed via the standard.

### 2.2. Content of Polyphenolic Compounds and Polymeric Procyanidins

Polyphenols are a very important group of secondary metabolites because they exhibit a wide range of health benefits and biological activities [41]. A statistical test on one-factor analysis indicated a significant effect (*p* < 0.05) of research plant parts on the total polyphenol content (TPC). The average TPC in the *S. palustris* L. was 6090 mg/100 g d.m. (Table 2). The highest amount of secondary metabolites were measured in the leaves (9252 mg/100 g d.m.), and this value was 1.9 and 5.7 times higher than in the stems and roots.

The main group of the analyzed polyphenols detected in the flowers, leaves, stems, and roots of *S. palustris* were hydrolysable tannins (constituting an average of 82.9% of all polyphenolic compounds) > flavones (8%) > flavan-3-ols (monomers and polymers; 5%) > phenylethanoid glycosides (3.3%) > phenolic acids, anthocyanins, and flavanols (<0.8%). Compared with other *Stachys* species, hydrolysable tannins were also a predominant group detected in *S. cretica* ssp. *anatolica* [42]. Nonetheless, higher TPC detected in the flowers and leaves is because these organs actively metabolize these compounds during photosynthesis [43,44].

A similar trend for TPC was noted in the studies on different parts of *Astragalus macrocephalus* subsp. *finitimus* [45]. In the *S. palustris* L. isolated from Hungary and France, the TPC was 17,630 and 24,980 mg GAE/100 g d.m., respectively [2], and was 1.8 and 2.5 times higher than in the leaves of *S. palustris* L. from Poland. In turn, the TPC of our extracts were significantly higher than those reported for other *Stachys* species (ranging from 430 mg GAE/100 g d.m. in *S. trinervis* to 4450 mg GAE/100 g d.m. in *S. fruticulosa*) from Iran by Khanavi et al. [46]. According to Oracz et al. [47], TPC may be influenced by many factors, e.g., origin, soil, weather conditions, analytical method, and the preparation of test samples. Carev et al. [42] reported that methanol extracts of *S. cretica* ssp. *anatolica* from Turkey showed 4330 mg/100 g d.m. TPC assessed by LC–ESI–MS/MS and 1.6 times lower TPC by spectrophotometric method [42]; the results were significantly lower than those reported in the flowers and leaves noted in our work. Likewise, during the analysis of *S. cretica ssp. Vacillans,* the record showed 3604 mg/100 g d.m. TPC using the HPLC system, and this value was 2.3 times lower compared to the spectrophotometric method [41]. On the other hand, the total concentration of TPC isolated from stems of *S. officinalis* L. were 6120 mg GAE/100 g dry extract [33], 1610–3330 mg GAE/100 g [48], which were 1.2 times higher and 2 times lower compared to stems of *S. palustris* L. The total amount of bioactive compounds extracted from leaves and roots of *Stachys officinalis* L. from Czech Republic were, respectively, 7495–8050 mg GAE/100 g d.m. and 2286–3164 mg caffeic acid/100 g d.m. (depending on the cultivar tested) [49]. TPC in the *S. recta* was 2050 mg/100 g d.m. and was around 4.8 times lower than in the leaves of *S. palustris* L. [25].

The most abundant group of tested parts of *S. palustris* L. contained a range of 1083 to 7945 mg/100 g d.m. for roots and flowers, respectively. The total tannins content identified in the different organs of *Calluna vulgaris* L. Hull in flowering time were 5, 4.3, and 3 times lower than our data for flowers, leaves, and stems [43]. In addition, the predominant compounds among 40 hydrolysable tannins were vescalagin and cocciferin d2 constituting 21% and 19%, respectively, in the leaves and 37% and 21% in the roots. This was also confirmed in the study by Esposito et al. [8] and Singh et al. [9].

The concentration of the second numerous group [42] ranged from 138.9 in the flowers to 1406.0 mg/100 g d.m. in the leaves. The most abundant compound in the flowers were luteolin 6-C-galactoside and 4′-*O*-methylisoscutellarein-glucoside-rhamnoside (constituting 33 and 26% of all flavones); in the leaves it was luteolin 6-C-galactoside and apigenin 6-C-glucoside (constituting 33 and 28%); in the stems it was luteolin 6-C-glucoside and apigenin 6-C-glucoside (constituting 33 and 27%); in the roots it was isoscutellarein-acetylallosyl-(glucopyranoside)apiose (constituting 55%). In turn, in studies by Carev et al. [42] the major compound in *S. cretica* ssp. *anatolica* was apigenin-7-glucoside and this was 2540 mg/100 g d.m. The total of flavonoids as quercetin equivalent in *S. tmolea* [50] and in *S. cretica ssp. vacillans* [41] were 500 and 5010 mg/100 g; in *Stachys cretica subsp. kutahyensis* there was 4020 mg/100 g extract [51]. In addition, apigenin and luteolin were reported to reveal a high anxiolytic potency in rats [52] and have high anticancer, antioxidant, and anti-inflammatory activities [50,53].

The next quantitatively important group was flavan-3-ols, including (−)-epicatechin as a monomer (constituting 5% of all flavan-3-ols) and polymeric procyanidins (PP; constituting 95%). The average amount of (−)-epicatechin in measured parts of the plant was 12.6 mg/100 g d.m. and PP—254.3 mg/100 g d.m. The (−)-epicatechin of measured parts may be organized in the following sequence: flowers > stems > leaves, and for PP: leaves > flowers > roots > stems. A similar trend for PP was noted in the different tested organs of *Rumex crispus* L. and *Rumex obtusifolius* L. [6]. The total of catechins as catechin equivalent in *Stachys marrubiifolia viv*. the leaf was 40 mg/100g extract [54]. The lowest amount of flavan-3-ols was detected in the stems and also confirmed by Feduraev et al. [6], probably by the inside metabolic action of the tissues and cells and the molecular constitution of the exudate carried by the phloem channels [6]. In addition, the alkaline solution of the central cavity of stems is exposed to oxidation PCs, including flavan-3-ols [6].

Moreover, the most opulent in PhGs were the roots, and the content was 209.5 mg/100 g d.m., and this amount was an average of 34 times higher than in the rest parts of *S. palustris* L. The main compound detected in the roots was isoverbascoside (verbascoside). This was also confirmed in the research on *S. cretica ssp. anatolica* by Carev et al. [47], on *S. cretica* subsp. *mersinaea* by Bahadori et al. [41], on *S. tmolea* by Elfalleh et al. [50], and on *Stachys cretica subsp. kutahyensis* [51]. During the measurement of *S. recta*, the content of PhGs was 607 mg/100 g d.m. and the concentration was around three times higher than in the stems of *S. palustris* L. [25]. In addition, the biological activity of verbascoside was confirmed, such as, for example, anti-tumor, anti-inflammatory, anti-radical, and anti-thrombotic effects [50].

In turn, the phenolic acids were the most abundant in the flowers compared to the rest parts of *S. palustris* L. tested, and their amount was 2.2, 3, and 8 times higher compared to the rest fractions. The total content of phenolic acids measured in the *S. recta* was 709 mg/100 g d.m. [25] and was significantly higher than our results. The anthocyanins, and flavonols were noted in the flowers. These compounds present less than 1% of all PCs in the measured extract of *S. palustris* L. The total of anthocyanins and flavonols as quercetin and cyanidin 3-glucoside equivalent in the *Stachys marrubiifolia viv*. leaves were 537 and 70 mg/100 g extract [54]. According to Bahadori et al. [41], the phenolic acid content measured in *S. cretica* subsp. *mersinaea* was 401 mg/100 g extract; in *S. tmolea* was 118 mg/100 g [50]. In turn, anthocyanin compounds, in addition to the health benefits, are responsible for the color of the raw material. According to the analysis, the flowers are the darkest and red with the addition of dark yellow color (Figure S1). In addition, the NAI index indicates an intense dark red pigment located in the flowers. On the other hand, the type of anthocyanins identified indicates a reddish-purple color. Of course, depending on the pH, it can change [2,29]. This is consistent with the botanical color of the flowers of the plant under study [55]. The root also seems interesting because it showed a dark red color with the addition of dark yellow [55].

### 2.3. In Vitro Biological Activity

#### 2.3.1. Antiradical and Reducing Potential

The antioxidant properties of *S. palustris* L. was assessed as reducing power (FRAP assay) and radical scavenging activity (ABTS assay). Results indicate that significant differences were noted between parts of the research plant. Table 3 suggests that the flowers and leaves had the highest radical scavenging activities (18.5 and 15.6 mmol TE/g d.m. respectively) and the highest FRAP reduction potential (5.6 and 7.3 mmol TE/g d.m., respectively). The ability to scavenge synthetic ABTS radicals determined for the roots and stems was about 2 and 3.8 times and 4.4 and 4.5 times weaker compared to the flowers and the leaves. Similar conclusions were found for the iron (III) reduction capacity, which was 5.3 and 18.5 times and 4 and 14.3 times weaker compared to the flowers and the leaves.

The results indicate that the flowers and the leaves are more effective at eliminating excess reactive oxygen species (ROS) that cause oxidative stress in the body compared to the roots and the stems [41,45]. When assessing the reducing power in *S. anisochila*, *S. beckeana*, *S. plumosa*, and *S. alpina* spp. *Dinarica*, a value of 1.9, 1.8, 0.5, and 1.4 mmol Fe^2+^/g d.m. has been reported [56]. Likewise, significantly lower values of antioxidant activity for the FRAP test were noted for the areal part of *S. trinervis*, *S. byzantina*, *S. setifera*, *S. subaphylla*, *S. turcomanica*, *S. inflata*, *S. laxa*, *S. persica*, and *S. fruticulosa* [45]. In *S. tmolea* (the areal part), the antiradical activity and reducing power was 32.3 and 41.9 mg TE/g d.m., respectively [50]. In studies conducted by Benabderrahim et al. [51], the antioxidant activity measured by ABTS and FRAP assays in *S. cretica* subsp. *Kutahyensis* were 175.8 and 239.1 mg TE/g of extract. The results obtained by Bahadori et al. [41] in *Stachys cretica* subsp. *Mersinaea* were 292.7 and 236.4 mg TE/g of extract for ABTS and FRAP tests, respectively. Whereas, in the extracts of *S. cretica* ssp. *Anatolica* for antiradical activity and reducing power were 112.2 and 127.2 mg TE/g of extract [42]. Moreover, our obtained results were not directly comparable. Besides, the research by Saravanakumar et al. [57] and Yadav et al. [58] shows that the differences in the value of antioxidant activity may significantly depend on the purity of solvents and their polarity, extraction procedure, and fractionation methods.

#### 2.3.2. In Vitro Enzyme Inhibition

Digestive enzymes, such as pancreatic lipase, are involved in fat metabolism and support the digestion of dietary fats into fatty acids. A-glucosidase and α-amylase are engaged in the carbohydrate metabolism and break down complex sugars into monosaccharides and oligosaccharides [57,59]. We analyze different parts of the plant with the digestive enzyme inhibitory properties to evaluate potential anti-obesity and anti-diabetic properties (Table 3).

The higher pancreatic lipase enzyme inhibitory activity was reported for leaves of *S. palustris* L. In addition, the flowers and leaves noted the higher α-amylase and α-glucosidase enzyme inhibitory properties. The least ability to inhibit the activity of digestive enzymes was noted for the roots; the ability to inhibit the activity of α-amylase was on average 3.5 times weaker compared to the leaves and the flowers, while the α-glucosidase activity was 3 times weaker. The ability to inhibit pancreatic lipase activity was noted for both the roots and the flowers.

The higher enzyme (α-amylase and α-glucosidase) inhibitory activity was evaluated in *Stachys japonica* of methanol extract, and the result was 7.43 ug extract/ug acarbose equivalent (ACE) [57]. Whereas the methanol extract from *S. cretica* subsp. *Smyrnaea* [41,60], *S. cretica* subsp. *Mersinaea* [41,60], and *S. cretica* subsp. *Kutahyensis* [51] had α-amylase inhibitory activity at the level 61.5, 418.6, and 315.5 mg ACE/g extract, respectively [50]. Furthermore, the inhibitory activity against α-glucosidase for *Stachys cretica* subsp. *Mersinaea* was 734.5 mg ACE/g of methanol extract [41]. Another study indicated that the α-amylase inhibition activity for *S. iberica* subsp. *Iberica var. densipilosa* and *S. byzantina* was 219.5 and 200.1 mg ACE/g, respectively [61].

We conclude that the leaves and flowers of *S. palustris* L. have PP and PA compounds that show potentially high antidiabetic activities and may be used for the inhibition of these enzyme activities. To our knowledge, the inhibition of α-amylase and α-glucosidase activity had not been reported before for *S. palustris* L. extracts.

#### 2.3.3. Anti-Proliferative Activity

**Figure 1:**
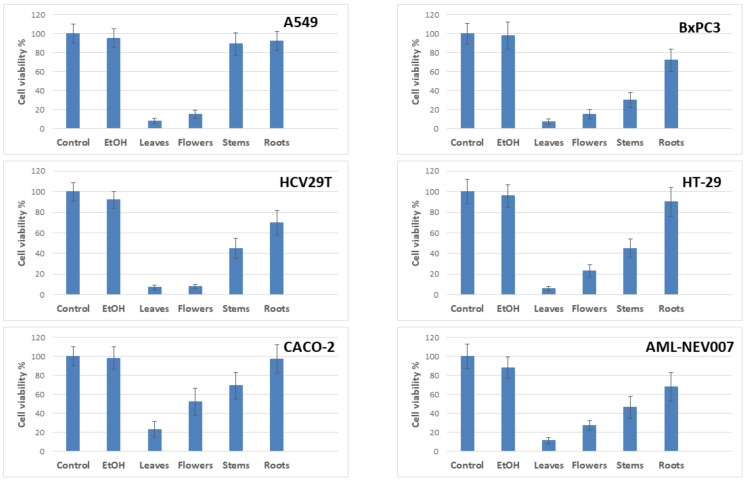
The viability of different cancer cells after treatment with 30% ethanolic extracts for 48 h. One 1:10 solid-to-liquid (S/L) ratio was used for all parts of the plant. Obtained extracts were diluted 30 times to final ethanol concentration 1%.

**Figure 2:**
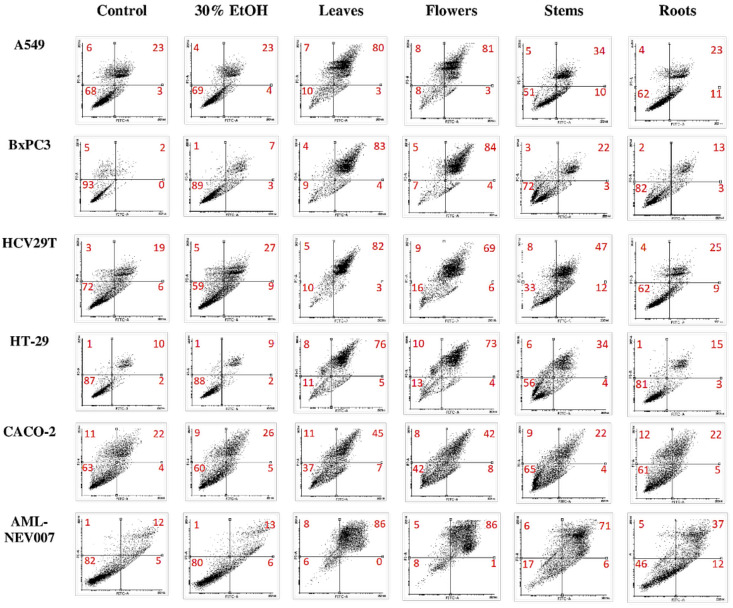
Induction of apoptosis after treatment for 48 h.

Anti-proliferative activity in the leaves, flowers, stems, and roots of *S. palustris* L. was tested in the A549 (lung adenocarcinoma), BxPC3 (pancreatic ductal adenocarcinoma), HT-29 and CACO-2 (colorectal adenocarcinoma), HCV29T (bladder cancer), and AML-NEV007 (acute myeloid leukemia) cell lines (Figure 1 and Figure 2). We chose tumors that are particularly resistant to chemotherapy and difficult to treat. Cells were treated with ethanol extract for 48 h, after which the MTS viability test and the analysis of the induction of apoptosis were performed.

The most significant results were obtained for leaves and flower extracts of *S. palustris* L. In particular, leaves extract markedly decreased the metabolic activity of all tested cell lines to 8–22%. Extract from the flower showed weaker inhibition effects (10–52% remaining activity). In addition, the roots and stems extracts were found weakest and reduced the viability to 30–90%. Diluted ethanol (1%) had no effect on cell lines.

We then tested the induction of apoptosis using Annexin V double staining and propidium iodide. As in the MTS test, the leaf and flower extracts significantly induced apoptosis in all tested cell lines in the range of 69–86%. The CACO-2 line was the most resistant, with the apoptosis level around 45%. A study by Kokhdan et al. [62] evaluated a methanol extract of *S. pilifera* against HT-29 cell line (colon adenocarcinoma) viability and demonstrated favorable inhibitory properties and significant anti-proliferative effects. It was also reported that *S. laxa* chloroform extract significantly prevented the proliferation of HT-29 and T47D (ductal carcinoma) cell lines, and the total extract of *S. subaphylla* also showed anti-proliferative properties against T47D cell line [63]. Furthermore, Haznagy-Radnai et al. [7], noted that some *Stachys* species, such as *S. palustris and S. recta,* stems in methanolic extracts displayed significant antiproliferative activity against cervix adenocarcinoma cells (HeLa); *S. germanica* flowers against breast adenocarcinoma (MCF-7) cells. In addition, Lachowicz et al. [11] noted that the methanolic extract of *Sanguisorba officinalis* L. leaves and flowers exhibited significant antiproliferative activity versus bladder cancer (HCV29T), colorectal adenocarcinoma (DLD-1), pancreatic ductal adenocarcinoma (BxPC3), and Jurkat cell lines. The obtained extracts contain a number of different substances that may have an additive or synergistic anti-proliferative effect on cancer cells. Many of these substances affect various intracellular pathways that depend on the type of cancer cells [64]. The externalization of Annexin V indicates the activation of caspases. Most likely, the process of apoptosis follows the classical mitochondrial pathway. Polyphenolic compounds are abundant in the leaves and flowers of *S. palustris* L. Luteolin, ellagic acid, and apigenin derivatives are known for their activating effects on the p53 transcription factor. They also affect the cell cycle and reduce the expression of the proapoptotic proteins from the Bcl-2 family [65]. Polyphenols have anti-inflammatory properties by inhibiting the activity of COX2 and NFKB, which is important in cancer [66]. Some natural compounds have exhibited synergism with established anticancer agents, and thus may reduce the side effects of chemotherapy. Further research is needed to identify active compounds and test their anti-cancer properties in appropriate in vivo models. The above results indicate that the anti-proliferative activity is mainly influenced by the part of the plant studied and material extraction, as well as the profile of PCs.

### 2.4. Multivariate Analysis

The results show a significant relationship between the high value of particular groups of the PCs and enzyme inhibitory activities, antioxidant, and antiproliferative activity, which was confirmed by the Pearson correlation coefficient. It is well known that PCs indicate numerous properties, including redox properties, and they, therefore, act as singlet oxygen quenchers and hydrogen donors, as well as exhibiting antioxidant activity [50]. Moreover, it is the synergistic effect between bioactive compounds in the tested material that provides antioxidant activity depending on, among others, their concentration [50]. Thus, a very strong correlation was noted between the antioxidant potential (for ABTS and FRAP assay, respectively) and the overall value of PCs (R^2^ 0.953 and 0.956), as well as individual groups of substances, such as hydrolysable tannins (R^2^ 0.967 and 0.901), polymeric procyanidins (R^2^ 0.853 and 0.978), and phenolic acids (R^2^ 0.792 and 0.685).

In addition, anthocyanins, flavan-3-ols, and flavonols had a stronger correlation with antiradical activity than FRAP assay (R^2^ 0.699, 0.684, 0.699, respectively), but flavones had a stronger correlation with reducing power than ABTS assay (R^2^ 0.700). A strong correlation between PCs and antioxidant assays was also reported by Bahadori et al. [41,60] and Khanaki et al. [45] in research concerning the analysis of *Stachys cretica* subsp. *Mersinaea*. On the other hand, the anthocyanins, flavan-3-ols, and flavonols showed negative correlation with BxPC3 cell line, and flavones noted negative correlation with α-glucosidase inhibitory activities.

Interestingly, PhGs, especially compounds identified in the roots, were the only group of PCs to show a negative correlation with enzyme inhibitory activities, a negative strong correlation with antioxidant activity, regardless of the method used, and antiproliferative activity in the all cells line.

The strongest relationships to inhibition of α-amylase activity were noted for hydrolysable tannins, flavones, and PP against the inhibition of α-glucosidase activity—hydrolysable tannins, anthocyanins, flavan-3-ols, phenolic acid, and flavonols; whereas the strongest correlations in inhibition of pancreatic lipase activity were noted for phenolic acid and PP. In turn, α-amylase inhibitory activities indicated a positive correlation with individual PCs, apart from *p*-coumaric acid and TPC (R^2^ −0.999) [41]. Furthermore, a strongly positive correlation was noted between the anti-proliferative potential (of all analyzed cancer cells line) and hydrolysable tannins, flavones, PP, and TPC; while the remaining groups of the polyphenolic compounds noted a positive correlation with cancer cells inhibitory activities, apart from PhGs.

**Figure 3:**
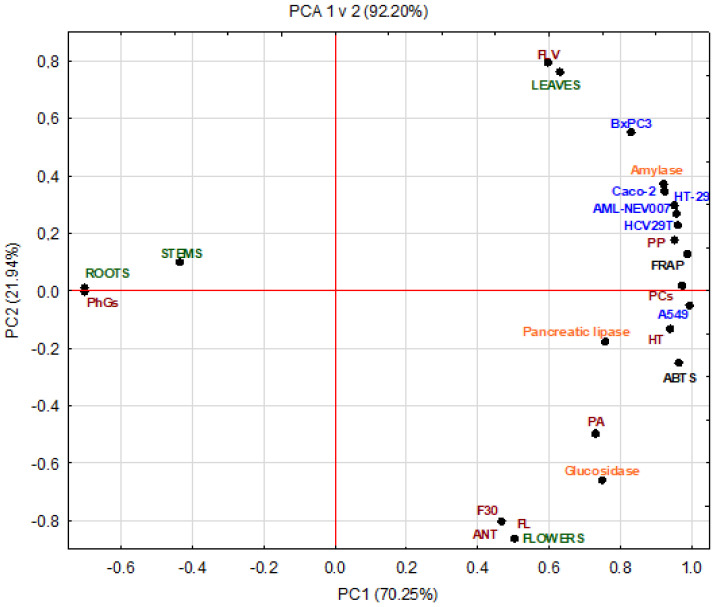
PCA of bioactive compounds and biological activities for all parts of *Stachys palustris* L.

Overall, the PCA results concerning different parts of plant extract data indicated a clear correlation with all PCs and anti-proliferative, anti-diabetic, anti-obesity, and antioxidant tests (Figure 3). The PCA detected two essential components that were responsible for 92.20% of data variance, including PC1 for 70.25%; while the second PC2 was only responsible for 21.94%. Figure 3 clearly shows the tested parts of *S. palustris* L. based on phytochemicals and antioxidant, anti-proliferative, anti-obesity, and anti-diabetic activities: stems and roots are found on the left of the plot, flowers and leaves are distributed on the right part. The obtained data indicates that the metabolites that distinguish the studied parts of the plant are the PCs and mainly hydrolyzed tannins and PP, which are found in high amounts in the leaves and flowers (PC1), and PhGs, which are especially present in the roots (PC2). Consequently, their type and concentration affect health-promoting properties. Therefore, the biological activities demonstrated a difference between parts of the plant. Besides, the difference in the profile and concentration of PCs in plant fractions is attributed to the difference in the morphological and anatomical structures and ongoing physiological processes [44,67]. The results of the PCA method showed similar results as the Pearson correlation.

## 3. Materials and Methods

### 3.1. Chemicals, Material and Instruments

Chemicals: Acetonitrile UHPLC, methanol, ascorbic acid, formic acid, methanol, ABTS (2,2′-azinobis(3-ethylbenzothiazoline-6-sulfonic acid), 6-hydroxy-2,5,7,8-tetramethylchroman-2-carboxylic acid (Trolox), 2,4,6-tri(2-pyridyl)-s-triazine (TPTZ), 2,2-Di(4-tert-octylphenyl)-1-picrylhydrazyl (DPPH), methanol, acetic acid, α-amylase from porcine pancreas, α-glucosidase from Rhizopus sp., lipase from porcine pancreas, 3,5-dini-trosalicylic acid, Antibiotic-Antimycotic Solution, and RPMI 1640 culture medium were purchased from Sigma-Aldrich (Steinheim, Germany). (−)-Epicatechin, ellagic acid, dicaffeic acid, kampferol-3-*O*-galactoside, malvidin-3-*O*-glucoside, delphinidin 3-*O*-glucoside, cyanidin-3-*O*-glucoside, apigenin, apigenin 6-C-glucoside, and luteolin were purchased from Extrasynthese (Lyon, France). DMEM culture medium with 10% FBS were purchased from Gibco (Thermo Fisher Scientific, Waltham, MA, USA), and MTS solution was purchased from Promega (Madison, WI, USA).

Material: Flowers, leaves, stems, and roots of *Stachys palustris* L. (~3 kg) were obtained from a private garden in Szczytna (53°33′46″ N 20°59′07″ E), Lower Silesia, Poland. The plant was collected randomly in August 2019 from different parts of the field (total area of cultivation is 1 ha). The root of the *Stachys palustris* L. plant by Professor Ireneusz Ochmian. The fresh flowers, leaves, stems, and roots were directly frozen at −25 °C, and then freeze-dried and crashed. The powders were kept frozen (−25 °C) until planned analysis around 2 weeks.

Instruments: Freeze-dryer (FreeZone 2,5, benchtop, A.G.A. Analytical, Warsaw, Poland), laboratory mill (RCMZ-800, Warsaw, Poland), Konica Minolta CM-700d spectrophotometer, ultra-performance reverse-phase liquid chromatography (UPLC-ESI-TQD-MS/MS) with Waters ACQUITY system (Waters, Milford, MA, USA), high-performance reverse-phase liquid chromatography with fluorescent detector (HPLC-FL) (Waters, Milford, MA, USA), centrifuge MPW-251 (MPW med. Instruments, Warsaw, Poland), Nicolet Evolution 300 spectrophotometer (Thermo, Watham, USA), Wallac 1420 VICTOR2 Plate Reader (PerkinElmer, Waltham, MA, USA).

### 3.2. Color Parameter

Color and shine of material were measured in a transmitted mode through Konica Minolta CM-700d spectrophotometer in 1 cm-thick glass trays. Measurements were conducted in CIE L*a*b* system, through a 10° observer type and D65 illuminant [68].

### 3.3. Polyphenolic Compounds (PCs) by UPLC-ESI-TQD-MS/MS and Procyanidin Polymers (PP) by the Phloroglucinolysis Method

The method procedure was applied according to the protocol shared by Kapusta et al. [69]. Profiles of polyphenolic compounds were analyzed using UPLC-PDA-ESI-TQD-MS/MS. Briefly, the separation was carried out using a BEH C18 column (100 mm × 2.1 mm i.d., 1.7 µm, Waters, Warsaw, Poland) that was kept at 50 °C. For the anthocyanins investigation, the following solvent system was applied: mobile phase A (2% formic acid in water, *v*/*v*) and mobile phase B (2% formic acid in 40% ACN in water, *v*/*v*). For other polyphenolic compounds, a lower concentration of formic acid was used (0.1%, *v*/*v*). The gradient program was set, as follows: 0 min 5% B, from 0 to 8 min linear to 100% B, and from 8 to 9.5 min for washing and back to initial conditions. The injection volume of samples was 5 µL (partial loop with needle overfill), and the flow rate was 0.35 mL/min. The following parameters were used for TQD: capillary voltage 3.5 kV, cone voltage, 30 V in positive and negative mode; the source was kept at 120 °C and the desolation temperature was 350 °C, con gas flow 100 L/h, and desolation gas flow 800 L/h. Argon was used as the collision gas at a flow rate of 0.3 mL/min. The profile compounds identification was based on specific PDA spectra, mass-to-charge ratio, and fragment ions obtained after collision-induced dissociation (CID). Quantification of compounds was achieved by the injection of solutions of known concentrations that ranged from 0.05 to 5 mg/mL (R^2^ ≤ 0.9998) as standards ((+)-catechin, 3,4-dicaffeoylquinic acid, luteolin 7-*O*-glucoside, apigenin 7-*O*-glucoside, kampferol-3-*O*-galactoside, ellagic acid, delphinidin 3-*O*-glucoside, cyanidin 3-*O*-glucoside, and malvidin 3-*O*-glucoside). All of the determinations were performed in duplicate and expressed as mg/L. Waters MassLynx software v.4.1 was used for data acquisition and processing. The analysis of polyphenolic compounds were assayed in triplicate, and described as mg per 100 g d.m.

The procyanidin polymers by the phloroglucinolysis method were applied according to the protocol shared by Lachowicz et al. [11]. The fractions of polymeric procyanidin were analyzed using HPLC-FL. Briefly, the separation was carried out using a BEH C18 RP column (2.1 × 5 mm, 1.7 μm; Waters Corporation, Milford, MA, USA) that was kept at 15 °C with gradient elution of solvent A as 2.5% acetic acid and solvent B as acetonitrile at a flow rate of 0.42 mL/min for a duration of 10 min (100 mm × 2.1 mm i.d., 1.7 µm, Waters) that was kept at 30 °C, and the fluorescence was recorded at the excitation and emission wavelengths at 278 and 360 nm. Quantification of compounds was made from procyanidin B2, (+)-catechin and (−)-epicatechin (R^2^ ≤ 0.9995) as standards. The degree of polymerization was calculated as the molar ratio of all flavan-3-ol units (phloroglucinol adducts + terminal units) to (−)-epicatechin and (+)-catechin, which correspond to terminal units. The analysis of polymeric procyanidins were assayed in triplicate, and described as mg per 100 g d.m.

### 3.4. In Vitro Biological Activity

#### 3.4.1. Extraction Procedure

The extraction was applied according to the protocol shared by Lachowicz et al. [11]. Briefly, 0.2 g of dry material was mixed with 7 mL of 80% of methanol in water and sonicated at 20 °C for 20 min, and then incubated at 4 °C for 24 h, and the next step was centrifuged. The supernatant was analyzed. The extractions method and all biological activities were assayed in triplicate.

#### 3.4.2. Antioxidant Activity

##### Antiradical Activity

The antiradical activity (ABTS) method was applied according to the protocol shared by Re et al. [70]. Briefly, 0.03 mL of extracted material mixed with 2.97 mL of ABTS solution and measured after 6 min at 734 nm using spectrophotometer. The result is described as mmol of Trolox equivalents (TE) per g d.m. (y = 33.64x + 2.68, R^2^ = 0.998).

##### Reducing Potency

The reducing potency (FRAP) method was applied according to the protocol shared by Benzie and Strain [71]. Briefly, 0.1 mL of extracted material with 0.9 mL of distilled water and 3 mL of ferric complex measured after 10 min of incubation at 593 nm using spectrophotometer. The result is described as mmol of Trolox equivalents (TE) per g d.m (y = 19.82x − 1.85, R^2^ = 0.999).

#### 3.4.3. Ability of α-Amylase, α-Glucosidase, Pancreatic Lipase Inhibitors

Ability of α-amylase, α-glucosidase inhibitors (anti-diabetic activity), and ability of lipase activity inhibitors (anti-obesity activity) effect of the extracted material were applied according to the protocol shared by Nakai et al. [72], Podsędek et al. [73], and Nickavar et al. [74]. Briefly, potato starch solution (0.2% (*v*/*v*)), the material extract, or phosphate buffer (0.1 M; pH 6.9; control) was mixed with α-amylase, and after incubation (37 °C, 10 min) the enzymatic reaction was stopped by the addition of HCl (0.4 M), the absorbance was read at 600 nm. For the α-glucosidase assay, the material extract was mixed with enzyme solution and incubated for 10 min. After that the reaction was initiated by *p*-nitrophenyl-α-D-glucopyranoside solution (5 mM), and incubated (37 °C, 20 min), and read the absorbance at 405 nm. For the pancreatic lipase assay, the material extracts were mixed with enzyme solution, and incubated (37 °C, 5 min). Then, methylumbelliferone solution (0.1 mM) was added, and incubated (37 °C, 20 min) and read at an excitation wavelength of 360 nm and at an emission wavelength of 460 nm. The value of the inhibitor, required to inhibit 50% of the enzyme activity, was defined as the IC_50_ value. The IC_50_ of the fruits tested was obtained from the line of the plot of the fruit concentration in 1 mL of reaction mixture versus the % inhibition.

#### 3.4.4. Antiproliferative Potency

##### Cell Lines and Cell Culture

The human cancer cell lines A549 (lung adenocarcinoma), BxPC3 (pancreatic ductal adenocarcinoma), HT-29, and CACO-2 (colorectal adenocarcinoma) and HCV29T (bladder cancer) were cultured in DMEM culture medium with 10% FBS (Gibco, Thermo Fisher Scientific, Walham, MA, USA) and Antibiotic Antimycotic Solution (Sigma-Aldrich, St. Louis, MO, USA). AML-NEV007 cell line (acute myeloid leukemia) was maintained in RPMI 1640 culture medium supplemented with 2 mM L-glutamine, 100 U/mL penicillin and 100 µg/mL streptomycin (Sigma-Aldrich, St. Louis, MO, USA), and 10% fetal bovine serum (FBS). All cell lines were cultured at 37 °C in a humidified atmosphere of 5% CO_2_. The cells were seeded at densities of 5 × 10^3^ cells/0.1 mL (0.32 cm^2^) for cell viability assay. All cell lines were obtained from the collection of the Institute of Immunology and Experimental Therapy, Polish Academy of Sciences, Wroclaw, Poland.

##### Determination of Cell Viability

The plant extract was applied according to the protocol shared by Lachowicz et al. [11]. Cell viability was assessed by the CellTiter 96 Aqueous One Solution Cell Proliferation Assay (Promega), according to the manufacturer’s protocol. Each treatment within a single experiment was performed in triplicate. Absorbance at 490 nm was recorded using a Wallac 1420 VICTOR2 plate reader (PerkinElmer, Waltham, MA, USA). Data were normalized to the untreated control.

##### Apoptosis Assay

Apoptosis was assessed by the Annexin V Apoptosis Detection Kit (Sigma-Aldrich, St. Louis, MO, USA), according to the manufacturer’s protocol. Briefly, the cells were incubated with all compounds for 48 h, next were stained with Annexin V-FITC (8 μg/mL) and PI (5 μg/mL) for 15 min at RT in the dark. The cells were washed with cold PBS (with Ca^2+^ and Mg^2+^) containing 2.5% FBS between the steps. Data were acquired using a FACSCalibur flow cytometer (Becton Dickinson, Franklin Lakes, NJ, USA) and analyzed using Flowing Software 2.5.1 (Perttu Terho, Turku, Finland). Apoptosis was quantified as a percentage of both Annexin V-positive and Annexin V/PI-double-positive cells.

### 3.5. Statistics

Statistical analysis included post-hoc Duncan’s multiple range test (*p* < 0.05) as one-way analysis. Principal component analysis (PCA) as the multivariate analysis were performed with Statistica 13.3 (StatSoft, Kraków, Poland).

## 4. Conclusions

Overall, the studies of PCs by the LC–PDA-ESI–TQD–MS/MS technique of different parts of *Stachys palustris* L. detected 89 polyphenolic compounds, including 40 hydrolysable tannins, 16 phenylethanoid glycosides, 4 anthocyanins, 1 flavan-3-ol, 6 phenolic acids, 1 flavonol, and 21 flavones. The profile and levels of these ingredients were conditional on the parts of *S. palustris* L. used; thus, in flowers, roots, leaves, and stems, there were 56, 55, 50, 49 compounds identified, respectively. The flavonols and anthocyanins were detected only in the flowers, while PhGs dominated the roots. In addition, the main compounds evaluated in the research were vescalagin, cocciferin d2, isoverbascoside (verbascoside), luteolin 6-C-glucoside, luteolin 6-C-galactoside, apigenin 6-C-glucoside, (−)-epicatechin, ellagic acid, and malvidin 3-*O*-diglucoside.

The highest amount of PCs was detected in the leaves, followed by the flowers, stems, and roots. The strongest antioxidant activity of the ABTS and FRAP assays for the leaves and flowers extracts were exhibited. In addition, the best digestive enzyme inhibition effect as a potential antidiabetic and anti-obesity activity. One of the most important features of leaf and flower extracts is their ability to induce cell death in various tumor cell lines. In turn, in this study, the roots and stems were statistically the weakest in terms of medicinal potential.

For these reasons, *S. palustris* L. leaves and flowers rich in natural antioxidants with high biological activity should be further examined as health-beneficial ingredients for functional food, special food, cosmetics, and/or medical and pharmaceutical industries. Further investigations are required to isolate and identify active compounds from leaves and flowers with anti-microbiological and antiproliferative effects and a wider range of antiproliferative effects, as well as the analysis of the bioavailability of compounds.

## Figures and Tables

**Table 1:** Characterization of polyphenolic compounds in *Stachys palustris* L. by LC–PDA-ESI–TQD–MS/MS.

Identified Compounds	Rr [min]	Δd [nm]	[M-H]^−^/MS-MS	Ref.
Hydrolysable tannins				
Grandinin	2.49	203	1065/975/931/301	[8,9,10,11,12,13,14,15,16,17,18,19]
Grandinin isomer	2.71	227	1065/975/931/301	[8,9,10,11,12,13,14,15,16,17,18,19]
Grandinin isomer	2.77	203	1065/975/931/301	[8,9,10,11,12,13,14,15,16,17,18,19]
Castalagin/vescalagin isomer	2.83	224	933/915/631/301	[8,9,10,11,12,13,14,15,16,17,18,19]
Vescalagin	2.93	224	933/915/631/613/569/467/301	[8,9,10,11,12,13,14,15,16,17,18,19]
Castalagin/vescalagin isomer	3.01	224	933/915/613/569/467/301	[8,9,10,11,12,13,14,15,16,17,18,19]
Castalagin/vescalagin (HHDP–NHTP–glucose) isomer	3.25	224	933/915/889/871/631/613/467/301	[8,9,10,11,12,13,14,15,16,17,18,19]
Pedunculagin isomer (diHHDP-glucose)	3.36	232	783/481/301	[8,9,10,11,12,13,14,15,16,17,18,19]
Castalagin/vescalagin isomer	3.43	204	933/915/889/631/301	[8,9,10,11,12,13,14,15,16,17,18,19]
Cocciferin d2 isomer (HHDP-NHTP-glucose-galloyldiHHDP-glucose)	3.59	224	933/915/631/301	[8]
Castalagin/vescalagin isomer	3.69	204	933/631/461/301	[8,9,10,11,12,13,14,15,16,17,18,19]
Pedunculagin isomer (diHHDP-glucose)	3.95	204	783/481/301	[8,9,10,11,12,13,14,15,16,17,18,19]
Castalagin/vescalagin isomer	4.02	208	933/915/631/301	[8,9,10,11,12,13,14,15,16,17,18,19]
Pedunculagin isomer (diHHDP-glucose)	4.24	230, 275	783/481/301	[8,9,10,11,12,13,14,15,16,17,18,19]
Castalagin/Vescalagin isomer	4.29	245	933/915/871/613/569/301	[8,9,10,11,12,13,14,15,16,17,18,19]
Casuarictin (galloyl-DiHHDP-glucose)	4.45	205	935/783/633/301	[8,9,10,11,12,13,14,15,16,17,18,19]
Cocciferin d2 isomer (HHDP-NHTP-glucose-galloyldiHHDP-glucose)	4.51	223	933/915/631/390/301	[8]
Chebulanin	4.54	205	651/481/463/337/319/275/169	[8,9,10,11,12,13,14,15,16,17,18,19]
Castalagin/vescalagin isomer	4.58	212/270	933/631/569/301	[8,9,10,11,12,13,14,15,16,17,18,19]
Chebulanin	4.60	206	651/481/463/337/319/275/169	[9,11,12,13,14,15,16,17,18,19]
Sanguiin H-10 isome (digalloyltriHHDPdiglucose)	4.87	315	1567/1265/1103/933/631/481/301	[20,21]
Castalagin/vescalagin isomer	4.93	216	933/633/481/301	[8]
Castalagin/Vescalagin isomer	5.05	276/353	933/915/871/631/613/467/301	[8]
Casuarinin (diHHDP-galloyl-glucose)	5.18	217	935/783/633/481/301	[8,9,10,11,12,13,14,15,16,17,18,19]
Castalagin/vescalagin isomer	5.33	220	933/783/633/434/301	[8]
Pedunculagin (diHHDP-glucose)	5.43	313	783/707/633/481/301	[8,9,10,11,12,13,14,15,16,17,18,19]
Sanguiin H-10 isomer (digalloyltriHHDPdiglucose)	5.51	240	1567/783/631/481/301	[20,21]
Roburin E	5.63	230	1064/301	[8,9,10,11,12,13,14,15,16,17,18,19]
Vescalagin isomer	5.91	218	933/631/467/301	[8,9,10,11,12,13,14,15,16,17,18,19]
Castalagin isomer	6.16	222	933/631/467/301	[8,9,10,11,12,13,14,15,16,17,18,19]
Geraniin isomer	6.25	209	951/933/633/301/257	[8,9,10,11,12,13,14,15,16,17,18,19]
Casuarinin/potentilin (galloyl-diHHDP-glucose)	6.51	279	935/783/633/301	[8,9,10,11,12,13,14,15,16,17,18,19]
Tellimagrandin I (digalloyl-HHDP-glucose)	6.53	218/277	785/615/483/301	[8,9,10,11,12,13,14,15,16,17,18,19]
Pentagalloyl-glucose	6.95	280	939/787/769/617/599/447	[8,9,10,11,12,13,14,15,16,17,18,19]
Tellimagrandin I isomer (digalloyl-HHDP-glucose)	6.99	218/277	785/615/483/301	[8,9,10,11,12,13,14,15,16,17,18,19]
Trigalloyl-HHDP-glucose	7.07	280	937/767/635/465/301	[8,14]
Trigalloyl-HHDP-glucose	7.74	280	937/767/635/465/301	[8,14]
Trigalloyl-HHDP-glucose	7.80	281	937/767/635/465/301	[8,14]
Trigalloyl-HHDP-glucose	7.93	281	937/767/635/465/301	[8,14]
Chebulagic acid (galloyl-chebuloyl-HHDP-glucose)	8.00	220/272	953/785/633/463/337/301/169	[14]
Phenylethanoid glycosides				
Echinacoside	6.27	329	785/623/461/161	[22,23]
Betonyoside E	6.32	326	785/639//621/609/193/161	[22,23]
Stachysoside A	6.38	272	755/623/461/593/179/161	[24]
B-OH-Forsythoside B methylether	7.29	330	785/755/623/347/161	[22,23]
Stachysoside A	7.41	330	755/623/593/461/179/161	[24]
Isoacteoside (isoverbascoside)	7.60	330	623/461/161	[2,25,26,27]
B-OH-Forsythoside B methylether	7.80	323	785/755/623/347/161	[22,23]
Forsythoside B isomer	7.91	330	755/623/607/593/461/161	[22]
Forsythoside B isomer	7.97	325	755/623/593/461/161	[22]
Forsythoside B isomer	8.07	326	755/623/461/447/161	[24]
Cistanoside A	8.22	328	799/637/623/475/315	[22,23]
Alyssonoside	8.34	329	769/593/575/447/315/161	[24]
Alyssonoside isomer	8.59	329	769/593/575/447/315/161	[24]
Martynoside	9.34	283	651/475/457/328/161	[24]
Samioside	10.02	329	755/593/461/315/161	[24]
Leucoseptoside A	10.29	313	637/461/315/193/175/161	[24,28]
Stachysoside E	10.43	319	669/625/583/380/264	[24]
Anthocyanins				
Delphinidin 3-*O*-glucoside	3.65	520	465/303	[29]
Malvidin 3-*O*-diglucoside	4.51	525	665/493/331	[29]
Cyanidin 3-*O*-glucoside	5.06	517	449/287	[29]
Malvidin 3-*O*-acetylglucoside	6.00	525	535/331	[29]
Flavan-3-ols				
(-)Epicatchin	4.83	280	289	[8]
Phenolic acid				
Ellagic acid glucoside	5.74	370	463/301	[25]
Ellagic acid pentoside	6.05	254/362	433/301	[25]
Ellagic acid	7.52	255/365	301	[25]
3,4-dicaffeoyl quinic acid	8.91	324	515/353/191/179	[25]
3,5-dicaffeoyl quinic acid	9.04	324	515/353/191/179	[25]
4,5-dicaffeoyl quinic acid	11.07	324	515/353/191/179	[25]
Flavonols				
Kaempferol hexose glucuronide	10.85	343	623/285	[30]
Flavones				
Chrysoeriol acetyl-allopyranosyl-glucopyranoside	5.82	269/332	677/299	[31,32]
Luteolin-6-C-galactoside	6.59	269/349	447/357/327/297/285	[33,34]
Luteolin-6-C-glucoside	6.74	267/347	447/357/327/299/285	[33,34]
Apigenin-6-C-galactoside	7.29	268/336	431/341/311/283/269	[35]
Apigenin-6-C-glucoside	7.43	269/336	431/341/311/283/269	[35]
Apigenin 7-*O-β-D*-(6-*p*-coumaroyl)-glucopyranoside	8.26	325	577/432/407/269	[31,36]
Apigenin acetyl-allosyl-glucoside	9.01	332	635/269	[26]
Chrysoeriol 7-*O*-acetylallosylglucoside	10.84	311	667/299	[25]
4′-O-methylisoscutellarein-diacetyl-allosyl-glucopyranoside	8.62	346	707/299	[25,26]
4′-O-methylisoscutellarein-acetyl-allosyl-glucopyranoside	8.87	329	665/485/299	[25,26]
Isoscutellarein-acetylallosyl-(glucopyranoside)apiose	9.19	276/330	651/429/285	[25,26]
Isoscutellarein-acetylallosyl-(glucopyranoside)apiose isommer	9.32	326	651/637/429/285	[25,26]
Isoscutellarein-acetylallosyl-(glucopyranoside)apiose isommer	9.44	329	651/607/429/285	[25,26]
Isoscutellarein-acetylallosyl-(glucopyranoside)apiose isommer	9.49	329	651/607/429/285	[25,26]
Isoscutellarein-acetylallosyl-(glucopyranoside)apiose isommer	9.63	329	651/285	[25,26]
Isoscutellarein-acetylallosyl-(glucopyranoside)apiose isommer	9.72	329	651/285	[25,26]
Isoscutellarein-acetylallosyl-(glucopyranoside)apiose isommer	9.79	314	651/285	[25,26]
Isoscutellarein-acetylallosyl-glucopyranoside	9.85	328	651/285	[25,26]
Isoscutellarein- acetylallosyl-glucopyranoside isommer	10.10	329	651/285	[25,26]
4′-*O*-methylisoscutellarein-acetyl-allosyl-glucopyranoside isomer	11.23	277/305	665/299	[25,26]

Rt, retention time.

**Table 2:** Total polyphenol content in different parts of *Stachys palustris* L. [mg/100 g d.m.].

Polyphenolic Compounds	Flowers	Leaves	Stems	Roots
Hydrolysable Tannins (HT)				
Grandinin	172.25 ± 2.07a ^a^	76.69 ± 0.92b	62.67 ± 0.75c	32.34 ± 0.39d
Grandinin isommer	399.62 ± 4.80b	533.26 ± 6.40a	230.27 ± 2.76c	nd
Grandinin isommer	265.51 ± 3.19a	nd ^b^	71.98 ± 0.86c	120.43 ± 1.45b
Castalagin/vescalagin isomer	214.78 ± 2.58b	494.02 ± 5.93a	212.20 ± 2.55b	nd
Vescalagin	2370.75 ± 18.97a	1528.04 ± 12.22b	1236.86 ± 9.89c	403.8 ± 3.23d
Castalagin/vescalagin isomer	36.41 ± 0.44c	224.02 ± 2.69a	95.17 ± 1.14b	nd
Castalagin/vescalagin (HHDP–NHTP–glucose) isomer	500.80 ± 6.01a	129.82 ± 1.56c	220.30 ± 2.64b	nd
Pedunculagin isomer (diHHDP-glucose)	nd	nd	nd	78.36 ± 0.94a
Castalagin/vescalagin isomer	945.11 ± 1.89b	1041.14 ± 2.08a	617.74 ± 1.24c	51.56 ± 0.62d
Cocciferin d2 isomer (HHDP-NHTP-glucose-galloyldiHHDP-glucose)	1552.66 ± 18.63a	1388.25 ± 16.66b	867.36 ± 10.41c	230.08 ± 2.76d
Castalagin/vescalagin isomer	636.42 ± 7.64b	990.62 ± 11.89a	572.37 ± 6.87c	37.70 ± 0.45d
Pedunculagin isomer (diHHDP-glucose)	63.94 ± 0.77b	80.22 ± 0.96a	27.38 ± 0.33c	2.59 ± 0.03d
Castalagin/vescalagin isomer	162.72 ± 1.95c	298.50 ± 3.58a	137.30 ± 1.65b	16.72 ± 0.20c
Pedunculagin isomer (diHHDP-glucose)	110.36 ± 1.32b	143.92 ± 1.73a	47.51 ± 0.57c	6.05 ± 0.07d
Castalagin/Vescalagin isomer	100.25 ± 1.20b	117.42 ± 1.41a	57.71 ± 0.69c	13.61 ± 0.16d
Casuarictin (galloyl-diHHDP-glucose)	33.64 ± 0.40b	85.27 ± 1.02a	22.76 ± 0.27c	34.51 ± 0.41b
Cocciferin d2 isomer (HHDP-NHTP-glucose-galloyldiHHDP-glucose)	21.33 ± 0.26b	29.19 ± 0.35a	8.57 ± 0.10c	nd
Chebulanin	nd	nd	nd	2.62 ± 0.03a
Castalagin/vescalagin isomer	40.36 ± 0.48a	nd	nd	nd
Chebulanin	nd	nd	nd	12.15 ± 0.15a
Sanguiin H-10 isomer (digalloyltriHHDPdiglucose)	4.73 ± 0.06c	9.11 ± 0.11b	3.84 ± 0.05cd	18.68 ± 0.22a
Castalagin/vescalagin isomer	27.11 ± 0.33a	28.71 ± 0.34a	17.43 ± 0.21b	4.79 ± 0.06c
Castalagin/Vescalagin isomer	18.71 ± 0.22a	1.32 ± 0.02b	0.95 ± 0.01b	nd
Casuarinin (diHHDP-galloyl-glucose)	38.79 ± 0.47a	18.53 ± 0.22b	7.82 ± 0.09c	3.42 ± 0.04d
Castalagin/vescalagin isomer	18.55 ± 0.22a	15.14 ± 0.18b	8.00 ± 0.10c	5.39 ± 0.06d
Pedunculagin (diHHDP-glucose)	5.76 ± 0.07b	11.64 ± 0.14a	5.43 ± 0.07b	1.47 ± 0.02c
Sanguiin H-10 isomer (digalloyltriHHDPdiglucose)	8.20 ± 0.10b	45.22 ± 0.54a	3.68 ± 0.04c	nd
Roburin E	27.43 ± 0.33a	20.53 ± 0.25b	4.11 ± 0.05c	nd
Vescalagin isomer	5.48 ± 0.07c	8.96 ± 0.11a	6.31 ± 0.08b	nd
Castalagin isomer	26.95 ± 0.32a	14.71 ± 0.18b	4.85 ± 0.06c	2.54 ± 0.03d
Geraniin isomer	1.63 ± 0.02b	5.28 ± 0.06a	4.57 ± 0.05a	nd
Casuarinin/potentilin (galloyl-diHHDP-glucose)	nd	1.39 ± 0.02b	3.85 ± 0.05a	1.59 ± 0.02b
Tellimagrandin I (digalloyl-HHDP-glucose)	5.38 ± 0.06a	nd	nd	2.32 ± 0.03b
Pentagalloyl-glucose	1.83 ± 0.02a	nd	nd	nd
Tellimagrandin I isomer (digalloyl-HHDP-glucose)	5.28 ± 0.06a	nd	nd	nd
Trigalloyl-HHDP-glucose	8.55 ± 0.10a	4.79 ± 0.06b	1.94 ± 0.02c	nd
Trigalloyl-HHDP-glucose	6.40 ± 0.08a	1.30 ± 0.02b	0.75 ± 0.01c	nd
Trigalloyl-HHDP-glucose	6.23 ± 0.07a	3.23 ± 0.04b	0.61 ± 0.01c	nd
Trigalloyl-HHDP-glucose	25.87 ± 0.31a	nd	nd	nd
Chebulagic acid (galloyl-chebuloyl-HHDP-glucose)	75.04 ± 0.90a	3.33 ± 0.04b	1.64 ± 0.02c	nd
Phenylethanoid glycosides (PhG)				
Echinacoside	nd	nd	nd	1.11 ± 0.01a
Betonyoside E	1.53 ± 0.02a	1.44 ± 0.02a	0.57 ± 0.01bc	1.10 ± 0.01b
Stachysoside A	6.62 ± 0.08a	nd	nd	nd
B-OH-Forsythoside B methylether	nd	nd	nd	15.01 ± 0.18a
Stachysoside A	nd	nd	nd	30.72 ± 0.37a
Isoacteoside (isoverbascoside)	nd	nd	nd	114.26 ± 1.37a
B-OH-Forsythoside B methylether	nd	nd	nd	1.04 ± 0.01a
Forsythoside B	nd	nd	5.76 ± 0.07b	10.84 ± 0.13a
Forsythoside B	nd	nd	nd	1.75 ± 0.02a
Forsythoside B isomer	nd	nd	nd	2.69 ± 0.03a
Cistanoside A	nd	nd	nd	1.33 ± 0.02a
Alyssonoside	nd	nd	nd	13.05 ± 0.16a
Alyssonoside isomer	nd	nd	nd	12.45 ± 0.15a
Martynoside	nd	nd	nd	4.17 ± 0.05a
Samioside	nd	nd	nd	0.92 ± 0.01a
Leucoseptoside A	nd	1.76 ± 0.02a	nd	nd
Stachysoside E	nd	1.70 ± 0.02a	nd	nd
Anthocyanins (ANT)				
Delphinidin 3-*O*-glucoside	3.36 ± 0.04a	nd	nd	nd
Malvidin 3-*O*-diglucoside	9.99 ± 0.12a	nd	nd	nd
Cyanidin 3-*O*-glucoside	3.07 ± 0.04a	nd	nd	nd
Malvidin 3-*O*-acetylglucoside	3.57 ± 0.04a	nd	nd	nd
Flavan-3-ols (F3O)				
(-)-Epicatchin	31.95 ± 0.38a	4.81 ± 0.06c	13.69 ± 0.16b	nd
Phenolic acid (PA)				
Ellagic acid glucoside	nd	4.24 ± 0.05a	0.71 ± 0.01b	nd
Ellagic acid pentoside	5.35 ± 0.06a	3.56 ± 0.04b	0.83 ± 0.01c	0.84 ± 0.01c
Ellagic acid	48.45 ± 0.58a	26.35 ± 0.32b	7.18 ± 0.09c	3.16 ± 0.04d
3,4-dicaffeoyl quinic acid	0.64 ± 0.01c	1.41 ± 0.02b	0.38 ± 0.00c	21.85 ± 0.26a
3,5-dicaffeoyl quinic acid	1.98 ± 0.02b	2.21 ± 0.03a	1.16 ± 0.01c	2.03 ± 0.02b
4,5-dicaffeoyl quinic acid	0.89 ± 0.01a	0.25 ± 0.00a	0.30 ± 0.00a	0.18 ± 0.00a
Flavonols (FL)				
Kaempferol hexose glucuronide	7.36 ± 0.09a	nd	nd	nd
Flavones (FLN)				
Chrysoeriol acetyl-allopyranosyl-glucopyranoside	nd	3.86 ± 0.05a	3.29 ± 0.04a	1.02 ± 0.01b
Luteolin-6-C-galactoside	22.57 ± 0.27c	462.91 ± 5.55a	72.75 ± 0.87b	0.46 ± 0.01d
Luteolin-6-C-glucoside	45.72 ± 0.55c	267.68 ± 3.21a	40.74 ± 0.49b	nd
Apigenin-6-C-galactoside	17.59 ± 0.21c	237.74 ± 2.85a	39.72 ± 0.48b	nd
Apigenin-6-C-glucoside	14.16 ± 0.17c	394.48 ± 4.73a	65.50 ± 0.79b	nd
Apigenin 7-*O-β-D*-(6-*p*-coumaroyl)-glucopyranoside	1.39 ± 0.02c	2.70 ± 0.03a	1.52 ± 0.02b	nd
Apigenin acetyl-allosyl-glucoside	nd	nd	nd	3.72 ± 0.04a
Chrysoeriol 7-*O*-acetylallosylglucoside	nd	2.01 ± 0.02a	nd	nd
4′-O-methylisoscutellarein-diacetyl-allosyl-glucopyranoside	36.59 ± 0.44a	31.61 ± 0.38b	17.67 ± 0.21c	nd
4′-O-methylisoscutellarein-acetyl-allosyl-glucopyranoside	nd	nd	nd	5.97 ± 0.07a
Isoscutellarein-acetylallosyl-(glucopyranoside)apiose	0.37 ± 0.00c	2.96 ± 0.04b	0.80 ± 0.01c	93.21 ± 1.12a
Isoscutellarein-acetylallosyl-(glucopyranoside)apiose isommer	nd	nd	nd	2.91 ± 0.03a
Isoscutellarein-acetylallosyl-(glucopyranoside)apiose isommer	nd	nd	nd	18.45 ± 0.22a
Isoscutellarein-acetylallosyl-(glucopyranoside)apiose isommer	nd	nd	nd	11.22 ± 0.13a
Isoscutellarein-acetylallosyl-(glucopyranoside)apiose isommer	nd	nd	nd	3.58 ± 0.04a
Isoscutellarein-acetylallosyl-(glucopyranoside)apiose isommer	nd	nd	nd	4.42 ± 0.05a
Isoscutellarein-acetylallosyl-(glucopyranoside)apiose isommer	nd	nd	nd	1.13 ± 0.01a
Isoscutellarein-acetylallosyl-glucopyranoside	nd	nd	nd	5.18 ± 0.06a
Isoscutellarein-acetylallosyl-glucopyranoside isommer	nd	nd	nd	2.69 ± 0.03a
4′-*O*-methylisoscutellarein-acetyl-allosyl-glucopyranoside isomer	nd	nd	nd	14.21 ± 0.17a
Procyjanidyny polimery (PP)	336.61 ± 4.04b	444.87 ± 5.34a	102.29 ± 1.23d	133.55 ± 1.6c
Degree of polymerization (DP)	2.96c	2.07d	3.72b	4.74a
The total sum of phenolic compounds	8544.63b	9252.11a	4938.76c	1622.94d

^a^ Values that are expressed as the mean (n = 3) ± standard deviation and different letters (between morphological parts) within the same row indicate statistically significant differences by Duncan’s test (*p* < 0.05); ^b^ nd, not identified.

**Table 3:** In vitro biological activity.

Parts of Plant	α-Amylase [IC_50_ (mg/mL)]	α-Glucosidase [IC_50_ (mg/mL)]	Pancreatic Lipase [IC_50_ (mg/mL)]	ABTS [mmol TE/g d.m.]	FRAP [mmol TE/g d.m.]
Leaves	6.85 ± 0.11a ^a^	12.71 ± 0.20b	27.46 ± 0.44a	15.55 ± 0.25b	7.25 ± 0.12a
Flowers	8.14 ± 0.13b	11.20 ± 0.18a	46.23 ± 0.74c	18.49 ± 0.30a	5.57 ± 0.09b
Stems	16.43 ± 0.26c	19.09 ± 0.31c	38.90 ± 0.62b	7.81 ± 0.12c	1.37 ± 0.02c
Roots	26.34 ± 0.42d	34.81 ± 0.56d	47.94 ± 0.77c	4.10 ± 0.07d	0.39 ± 0.01d

^a^ Values that are expressed as the mean (n = 3) ± standard deviation and different letters (between morphological parts) within the same row indicate statistically significant differences (*p* < 0.05).

## Data Availability

Not applicable.

## Supplementary Materials

The following supporting information can be downloaded at: https://www.mdpi.com/article/10.3390/ph15070785/s1, Figure S1: LC-PDA-ESI-TQD-MS/MS chromatogram fragile of the *Stachys palustris* L. roots extract at 280 and 320 nm; Table S1: Parameter of color.
