# Supplementary material for: Flowers and Leaves Extracts of Stachys palustris L. Exhibit Stronger Anti-Proliferative, Antioxidant, Anti-Diabetic, and Anti-Obesity Potencies than Stems and Roots Due to More Phenolic Compounds as Revealed by UPLC-PDA-ESI-TQD-MS/MS
Source: Pharmaceuticals (Basel). 2022 Jun 23;15(7):785. doi: 10.3390/ph15070785 (PMC9317270; doi:10.3390/ph15070785)
Supplement: Supplementary file 1 [file pharmaceuticals-15-00785-s001.zip › pharmaceuticals-1720443-Supplementary Materials.pdf]

pharmaceuticals

MDPI

Article

Flowers and Leaves Extracts of *Stachys palustris* L. Exhibit Stronger Anti-Proliferative, Antioxidant, Anti-Diabetic, and Anti-Obesity Potencies than Stems and Roots Due to More Phenolic Compounds as Revealed by UPLC-PDA-ESI-TQD-MS/MS

Sabina Lachowicz-Wiśniewska [1,2,3,*], Anubhav Pratap-Singh [3], Ireneusz Kapusta [4], Angelika Kruszyńska [5], Andrzej Rapak [5], Ireneusz Ochmian [2], Tomasz Cebulak [4], Wioletta Żukiewicz-Sobczak [1] and Paweł Rubiński [1]

1 Department of Food and Nutrition, Calisia University, 4 Nowy Świat Street, 62-800 Kalisz, Poland; s.lachowicz-wisniewska@akademiakaliska.edu.pl (S.L.-W.), wiola.zukiewiczsobczak@gmail.com (W.Ż.-S.); p.rubinski@akademiakaliska.edu.pl (P.R.)
2 Department of Horticulture, West Pomeranian University of Technology in Szczecin, 71-434 Szczecin, Poland; ireneusz.ochmian@zut.edu.pl
3 Faculty of Land and Food Systems (LFS), The University of British Columbia, Vancouver Campus 213-2205 East Mall, Vancouver, BC V6T 1Z4, Canada; anubhav.singh@ubc.ca
4 Department of Food Technology and Human Nutrition, College of Natural Science, Rzeszow University, 4 Zelwerowicza Street, 35-601 Rzeszow, Poland; ikapusta@ur.edu.pl (I.K.); tomcebulak@gmail.com (T.C.)
5 Laboratory of Tumor Molecular Immunobiology, Ludwik Hirszfeld Institute of Immunology and Experimental Therapy, Polish Academy of Sciences, 53-114 Wroclaw, Poland; angelika.kruszynska@hirszfeld.pl (A.K.); andrzej.rapak@hirszfeld.pl (A.R.)
* Correspondence: s.lachowicz-wisniewska@akademiakaliska.edu.pl

Citation: Lachowicz-Wiśniewska, S.; Pratap Singh, A.; Kapusta, I.; Kruszyńska, A.; Rapak, A.; Ochmian, I.; Cebulak, T.; Żukiewicz-Sobczak, W.; Rubiński, P. Flowers and Leaves Extracts of *Stachys palustris* L. Exhibit Stronger Anti-Proliferative, Antioxidant, Anti-Diabetic, and Anti-Obesity Potencies than Stems and Roots Due to More Phenolic Compounds as Revealed by UPLC-PDA-ESI-TQD-MS/MS. *Pharmaceuticals* **2022**, *15*, x. https://doi.org/10.3390/xxxxx

Academic Editor(s): Célia Cabral

Received: 25 April 2022
Accepted: 20 June 2022
Published: date

Publisher's Note: MDPI stays neutral with regard to jurisdictional claims in published maps and institutional affiliations.

Copyright: © 2022 by the authors. Submitted for possible open access publication under the terms and conditions of the Creative Commons Attribution (CC BY) license (https://creativecommons.org/licenses/by/4.0/).

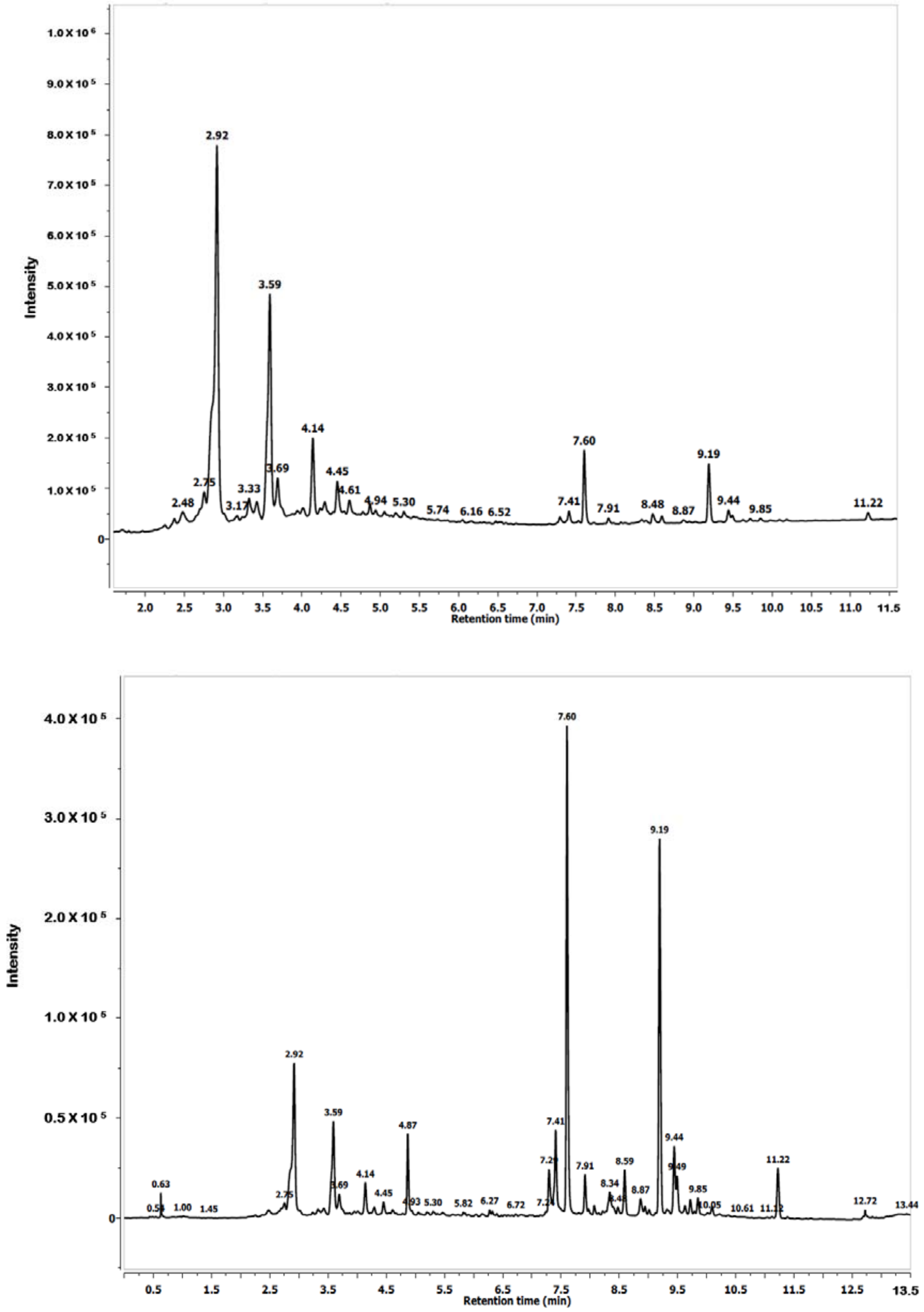

Figure S1. LC-DAD-ESI-TQD-MS/MS chromatogram fragile of the *Stachys palustris* L. roots extract at 280 and 320 nm.

Table S1. Parameter of colour.

Parameter of colour	L*(D65)	a*(D65)	b*(D65)	NAI	NDVI
Leaves	51.57±1.03[a]c	-50.81±1.02d	31.51±0.63a	-0.72±0.01d	0.76±0.02a
Flowers	40.82±0.82d	35.49±0.71a	13.01±0.26d	0.67±0.01a	0.50±0.01c
Stems	62.17±1.24b	-23.00±0.46c	25.68±0.51b	-0.41±0.01c	0.66±0.01b
Roots	60.70±1.21a	5.42±0.11b	21.64±0.43c	0.11±0.00b	-0.24±0.00d

[a] Values are expressed as the mean (n = 3) ± standard deviation and different letters (between morphological parts) within the same row indicates statistically significant differences ($p < 0.05$).
